# Using Human-Centered Design to Develop, Launch, and Evaluate a National Digital Health Platform to Improve Reproductive Health for Rwandan Youth

**DOI:** 10.9745/GHSP-D-21-00220

**Published:** 2021-11-29

**Authors:** Nicole Ippoliti, Mireille Sekamana, Laura Baringer, Rebecca Hope

**Affiliations:** a Youth Development Labs (YLabs), Kigali, Rwanda.

## Abstract

**Background:**

A lack of access to evidence-based, unbiased, and youth-friendly family planning and reproductive health (FP/RH) information and care limit young people's ability to prevent unplanned pregnancies and HIV and sexually transmitted infections. This threat-ens their health and is a significant cause of school drop-out, limiting young peoples’ well-being, future potential, and employment opportunities. To address these challenges facing youth, YLabs used an end-to-end human-centered design (HCD) approach to create CyberRwanda, a digital platform aiming to improve the health and livelihoods of adolescents (aged 12–19 years) in Rwanda.

**Designing for digital with youth:**

From 2016 to 2020, CyberRwanda was designed and piloted using an HCD approach in partnership with more than 1,000 youth, parents, teachers, and public and private health care providers. During the problem recognition phase, HCD revealed participants’ beliefs, behavioral preferences, and experiences as they relate to FP/RH specifically and their broader life experiences, motivations, and challenges. Several phases of analog, digital, and live prototyping with youth and key stakeholders were used to codesign, test, and refine the intervention for implementation.

**Results:**

CyberRwanda is a direct-to-consumer platform where adolescents can learn integrated, age-appropriate health, and skills-building information through edutainment behavior change stories and a robust frequently asked questions library, order health products online, and be linked to CyberRwanda’s network of private and public health care providers who have been trained to provide adolescent-friendly care.

**Implications for future research:**

The HCD process resulted in significant pivots to the design of the digital platform and the implementation model. Using HCD provided a structured methodology to combine technical FP/RH expertise and visual and product design expertise to codesign and iteratively develop a digital health intervention with and for Rwandan youth.

## Abstract (precis)

Human-centered design, done with attention to meaningful participation, equity, and accessibility, is an effective methodology to design digital health interventions with and for youth as it places their unique needs and motivations at the center of the design and helps to ensure usability, equity, and accessibility.

## Teaser

**Key Messages:**

- Using a human-centered design (HCD) approach throughout the design and adaptive implementation phase with youth and key stakeholders led to major pivots in the product, implementation, and evaluation design of a digital health product for Rwandan youth.
- While using an HCD process to develop CyberRwanda, it was critical to include a diverse population of young people and community stakeholders as both collaborators and leaders in a youth-driven design process.
- Prioritizing equity of access led to the design of interventions for users with and without their own devices and with varying levels of digital literacy and experience using digital platforms.

**Key Implications:**

- Organizations developing digital health products for young people should consider taking an end-to-end human-centered design approach for the product development and intervention model.
- With increasing interest and investment in digital health interventions, especially those supporting direct-to-consumer products, funders should support and encourage funding and time for codesign, development, and prototyping with the intended audience in both the design and implementation phases.
- HCD practitioners designing for youth need to ensure appropriate representation of diverse groups of young people, take extra care to address power differentials between young people and designers, and ensure they have robust safeguarding policies and practices.

Résumé en français à la fin de l'article.

## BACKGROUND

Adolescence and young adulthood are critical windows of opportunity to influence positive habit formation, behavior, and development. There is growing consensus among researchers, policy advocates, and practitioners worldwide that recognizes that interventions that are developed in partnership with youth are more likely to be effective at engaging young people resulting in greater impact on behavioral and biological outcomes of interest.^1^^,^^2^

Over the last decade, human-centered design (HCD) has emerged as a promising methodology to engage youth in the design and adaptation of solutions to better meet their needs. HCD is a creative problem-solving process used to develop interventions that center on the person or beneficiary. The HCD process provides a framework to design and iterate on interventions while taking into account desirability, feasibility, and viability.^3^

Preliminary data suggest that an HCD approach more successfully engaged Zambian adolescents with family planning (FP)/sexual and reproductive health (RH) services than standard care.^4^ In Kenya, a redesign of adolescent health services using HCD resulted in a 7-fold increase in the number of adolescents visiting health centers and a 70% increase in uptake of long-acting reversible contraceptives.^5^ There are forthcoming randomized controlled trials (RCTs) on HCD programs to improve access to RH and FP information and services for youth in Rwanda,^6^ to increase HIV self-testing in Tanzania,^7^ and to address provider bias toward youth in Burkina Faso, Pakistan, and Tanzania.^8^

Although rigorous documentation on using HCD to design global health interventions has increased, to date, there are limited documented examples of applying HCD throughout the entire health intervention-development cycle. This includes understanding the problem from the user’s perspective, informing the solution design, and implementing and measuring progress, all in partnership with the end user. Even fewer interventions have documented conducting this process in partnership with youth.

This article describes the HCD approach that Youth Development Labs (YLabs) used in Rwanda to codesign, pilot, evaluate, and design CyberRwanda, a digital behavior change intervention designed to improve the health and livelihoods of adolescents aged 12–19 years. CyberRwanda weaves together behavior change stories featured via webcomics, a robust frequently asked questions (FAQs) library, online ordering of health products, and a pharmacy/health facility locator. Combined, these features aim to deliver integrated, age- appropriate adolescent health, and economic empowerment information and linkage to quality youth-friendly services. CyberRwanda also trains pharmacists and nurses at participating health facilities on addressing provider bias, youth’s right to access health products in Rwanda, and voluntary FP/RH care.

CyberRwanda aims to deliver integrated, age-appropriate adolescent health, and economic empowerment information and linkage to quality youth-friendly services.

YLabs is a leading global design and research organization working to improve health and economic opportunity for young people aged 10–24 years. YLabs’ mission is to design, test, and advocate for youth-driven solutions that address the biggest challenges to young people’s health and economic opportunity, including sexual and reproductive health, HIV, and mental health. YLabs combines diverse disciplines to develop and measure innovative solutions with young people and their communities. Our team, including young people among the 16 countries where we work, brings expertise in adolescent health, behavioral science, mental health, impact evaluation, digital product design and development, marketing, and brand design.

This article documents process findings that will reveal how taking a youth-driven and youth-led design approach, as defined by YLabs (Table 1), played a critical role in understanding the needs of young people, brought forth innovation to traditional forms of information and service delivery, and highlighted the special considerations of designing with youth.^9^

**TABLE 1.** Distinctive Approaches to Designing With Youth

**Term**	**Definition**
Youth-led design	Young people make the decisions on which problem statements to focus on. They are facilitators and designers of the solution development process, either with or without guidance from an adult team.
Youth-driven design	Young people’s voices and perspectives drive the decisions about the interventions and approaches that will affect them and their communities. Young people are actively involved in the decision-making processes and lead key project activities.
Youth-centered design	Youth are the audience of focus for the intended intervention or approach. They participate in the design process by providing data, input, or feedback to an adult design team. Typically, they are not part of the team that decides on the intervention or proposed approach.

## FP/RH CONTEXT AMONG ADOLESCENTS IN RWANDA

In Rwanda, adolescents have limited access to high-quality FP/RH information and care to prevent unplanned pregnancy, HIV, and sexually transmitted infections. Despite a strong national commitment to reducing unplanned adolescent pregnancies, the adolescent fertility rate in Rwanda has not changed significantly between 2010 and 2018 from 40 live births to 39 live births (per 1,000 women aged 15–19 years).^10^^,^^11^ Among sexually active adolescents aged 15–19 years, nearly 50% are not using any form of contraception.^12^ Youth-friendly services remain limited, with only 13.6% of health facilities providing these services.^13^

With limited access to services and insufficient training of providers on adolescent-friendly care, youth who access FP/RH services often experience disproportionately lower-quality care and provider judgment. Adolescents also lack access to quality, adolescent-focused information. Although the Rwandan Ministry of Education has committed to providing comprehensive sexuality education in schools and has invested in training schools on the new curriculum, there is a lack of quality materials to support teachers in its delivery.

With rapidly increasing access to digital devices and the Internet for young people in sub-Saharan Africa, there has been a surge of interest and investment in digital approaches to support the delivery of quality FP/RH information and care for youth. Digital channels provide an opportunity to support youth, a population who are known to be early adopters of technologies, in learning about their health and take positive steps to access health services and products privately and on their own time.^3^^,^^14^ In a recent global survey of youth, with more than one-third of participants from sub-Saharan Africa, 62% already reported using technology for their own health needs, and 84% planned to use digital tools to track their health in the future.^2^ Digital interventions that are designed with attention to adolescents’ desire for autonomy, social connection, and agency have the potential to reach adolescents and young adults during critical neurodevelopmental windows of opportunity to influence positive habit formation, behaviors, and learning.^15^

The challenge of designing impactful digital health interventions is ensuring equity, access, and usability for new technology users, especially in low-resource settings. In Rwanda, digital usage is on the rise with mobile cellular subscriptions more than doubling between 2010 and 2019 to 76.5%, in line with pan-African trends.^16^ Current annual growth in active social media use is 20% and internet use is 8.8%.^17^ However, there are marked differences in access to phones by socioeconomic status, between rural and urban contexts, and between boys and girls.^18^^,^^19^ Among youth, there are persistent gender gaps in phone access and ownership and in the use of mobile money and e-commerce.^4^^,^^20^ Youth commonly share phones, which can adversely impact the level of privacy they experience in accessing digital health information and service. These considerations must be further explored when designing a digital intervention for young people to avoid designing only for urban or elite youth, thus exacerbating existing health inequities.^21^

The challenge of designing impactful digital health interventions is ensuring equity, access, and usability for new technology users, especially in low-resource settings.

## DESIGNING FOR DIGITAL WITH YOUTH

Between 2016 and 2019, CyberRwanda was developed using a youth-driven and youth-led design process with 1,074 Rwandan youth, caregivers, teachers, health care providers, and government stakeholders (Figure).

**FIGURE:**
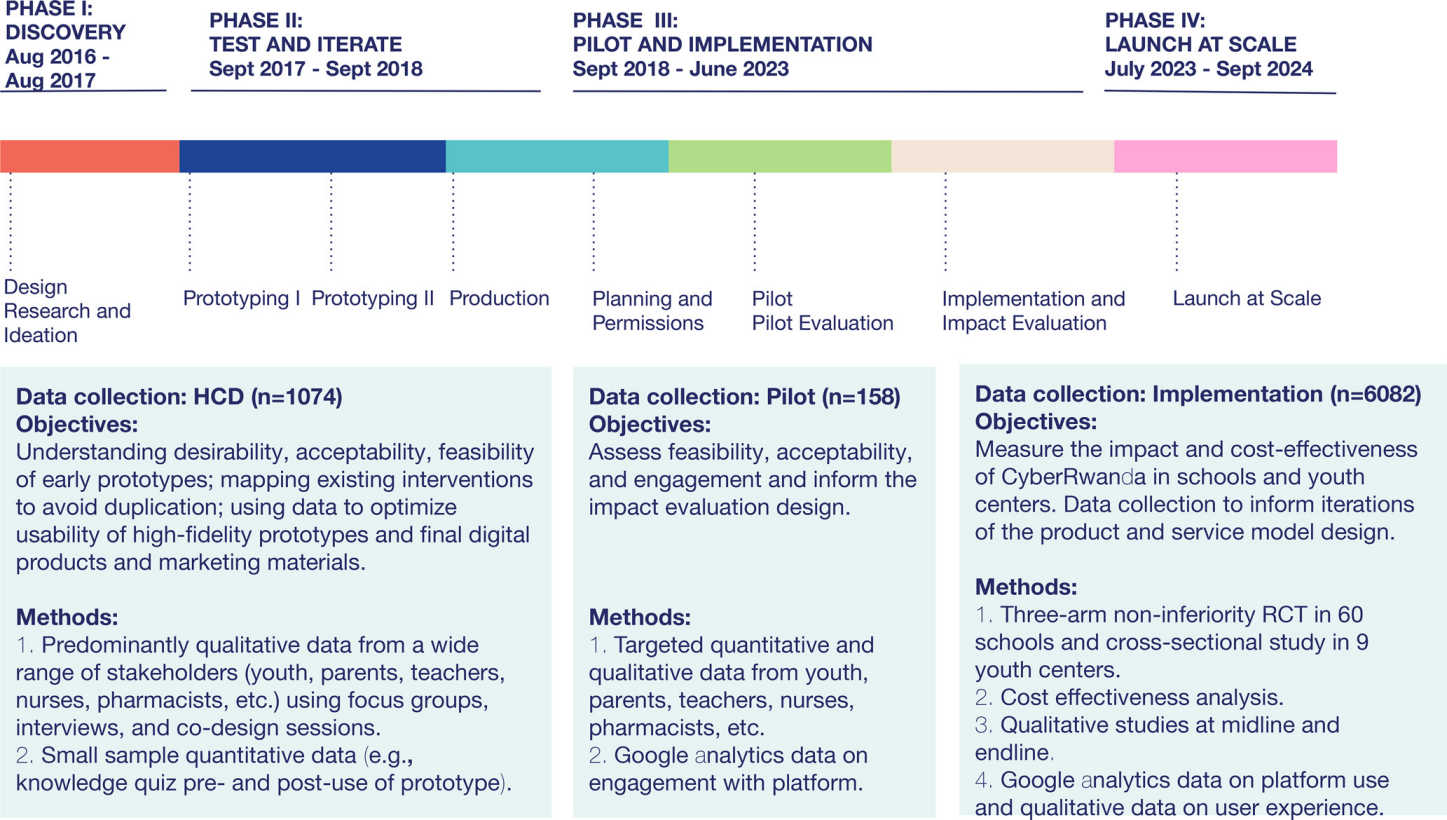
Overview of Phases of HCD Phases, Data Collection Objectives, and Methods Throughout the Design, Pilot, and Implementation of the CyberRwanda Project Abbreviations: HCD, human-centered design; RCT, randomized controlled trial.

The platform’s intended outcomes for adolescents aged 12–19 years were to:

1. **Improve knowledge:** Help adolescents gain employment skills, set future goals, and learn about FP/RH in a private, safe space
2. **Improve access:** Support adolescents’ ability to access FP/RH products and services by strengthening linkages to services
3. **Improve quality:** Improve providers’ knowledge on FP/RH and address provider bias to improve service quality for youth

We describe the methods used to design CyberRwanda and the populations engaged throughout the phases of design research, prototyping of initial and refined concepts, product and content development, pilot, and adaptive implementation and evaluation. Throughout the design and implementation process from 2016 to 2021, our team moved from youth-centered design to youth-driven, to a youth-led approach today (Table 1 provides definitions of terms), where youth members of the team led decision making on intervention design. This transition from youth-centered to youth-driven and youth-led approaches was supported through dedicated training of youth members in the following: how to conduct each phase of the HCD process, conducting user experience testing of digital prototypes, and ensuring the safeguarding and protection of youth participants. Additionally, all staff members (youth and adult) were trained in youth-driven or youth-led design principles. This includes how to recognize and address the power imbalances that exist between adult and youth team members and how to create opportunities for youth members to lead activities and/or cofacilitate with appropriate support and guidance.

Throughout the design and implementation process, our team moved from youth-centered design, to youth-driven, to a youth-led approach.

### Design Research

Design research is the primary stage in the HCD process that leverages qualitative and observational data to understand human behavior, latent needs, and the environmental factors or social norms that might contribute to the design of an intervention. The team began the design research phase (Table 2) with the key question of how to leverage technology to address 2 pressing and inextricably linked issues facing young people: teen pregnancy and unemployment, with young people as the primary users. This phase consisted of interactive codesign workshops, focus group discussions, and in-depth interviews with young people, health care providers in both public and private facilities and pharmacies, parents, government officials, and other critical stakeholders. Interviews were conducted by Rwandan youth designers, in Kinyarwanda with simultaneous English translation. During the HCD process, participants were purposely sampled in 8 districts (Gasabo, Nyarugenge, Kicukiro, Nyagatare, Ruhango, Huye, Musanze, Rwamagana) in rural, urban, and peri-urban contexts, to ensure diverse representation regarding socioeconomic status, gender, age, educational status, and social vulnerabilities (participants included former sex workers, insecurely housed youth, and domestic workers). A literature and policy review and landscape analysis of existing digital and youth-centered programs was also conducted to ensure that CyberRwanda’s focus was non-duplicative and aligned with national priorities.

**TABLE 2.** Selected Design Research Methods Used for CyberRwanda

**Method**	**Examples of How They Were Used for CyberRwanda**
Card sorting	Participants ranked cards with different images of people in order of preference (e.g., people they trust most with sensitive information or who they would ask for information on reproductive health). Additional prompts and discussion informed the initial design of the characters who deliver information in the webcomic.
Vignette cards	To explore provider knowledge and biases, pharmacists and clinic staff were asked to opine on which family planning methods and reproductive health services might be “appropriate” for different mock clients.
Role play	Role play was used extensively to understand taboos and stigma that youth held in discussing their reproductive health with others, including peers, providers, teachers, and parents, and prompt discussion on how to address these challenges. Dialogues also helped inform the style and tone of conversational content for the webcomic.
Codesign activities	Workshops gather audiences of focus to take part in the creative development process of brainstorming ideas for a specific design challenge and then bring them to life by creating rough representations of their solution idea. Youth were asked to develop paper prototypes of their own campaign addressing adolescent pregnancy in Rwanda to prompt further discussion on messaging, content, and digital and nondigital delivery channels (e.g., social media, magazines, and school lessons).
Surveys	Rapid, small sample surveys were used during design research to capture baseline knowledge, demographics, and technology usage among participants.
Journey mapping	Participants drew their journey maps, setting different life milestones and their expected timeline for their achievement.
Mystery clients	Mystery clients are trained community members who visit services or facilities in the role of the patient or client and report on their experience. Mystery clients were used to explore the service experience for young people requiring different products and services at pharmacies.
Observation	Observation was a critical method used to understand the level of privacy, dignity, and comfort for young people accessing health care services. In schools, observation of teachers, equipment, and classroom spaces helped us test assumptions around using school laptops to deliver CyberRwanda. Observations that existing laptops were not easy to use by untrained staff and did not afford privacy led to a pivot to instead use tablets in kiosks or school clubs.
Personas^a^	Personas were used to better define potential groups of users with shared characteristics regarding knowledge, needs, and access to technology.

^a^ Personas are adapted from World Health Organization (WHO). *Digital Implementation Investment Guide (DIIG): Integrating Digital Interventions Into Health Programmes.* WHO; 2020. https://apps.who.int/iris/rest/bitstreams/1303132/retrieve.

The design research phase enabled the team to rapidly assess the desirability of a digital intervention and potential barriers to access for urban/peri-urban youth. It also revealed young peoples’ and providers’ beliefs and behavioral drivers and barriers as they related to FP/RH. Journey mapping was used to understand topics and themes that youth found most interesting (Table 2). Young people mapped their various life goals and important milestones and by when they hoped to achieve them. The youth then listed resources and information they would need to achieve those goals at the desired age. This process provided important insights into a young person’s lived reality; mapping their everyday experiences, goals, motivations, and challenges enabled the design team to develop prototypes that integrated content and features that ultimately resonated with what young people found to be desirable and inspirational. Research with parents, teachers, and health care providers revealed how to develop prototypes that would be socially accepted and aligned with national priorities, revealed appropriate “entry points” when discussing sensitive topics, and unearthed pharmacists as potential secondary users.

**Figure:**
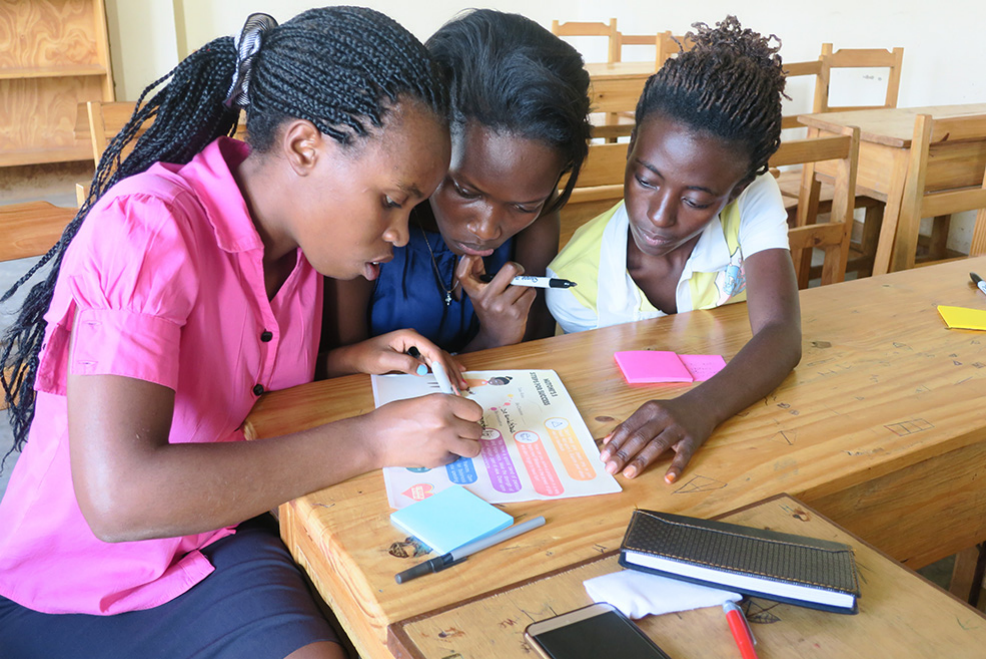
Conducting design research of reproductive health content with adolescent girls. © 2016 Laiah Idelson/YTH

In the design research phase, journey mapping was used to understand topics and themes that youth found most interesting.

### Prototyping

Using the learnings from the design research phase, multiple rough prototypes were tested with young people and pharmacists. Rough prototypes are quick, tangible, low-fidelity versions of a product or intervention used to gather rapid user feedback on the design, content, delivery channel, and experience. Table 3 shows selected methods used to share prototypes with participants and the methods of eliciting feedback. These rough prototypes not only enabled the team to test and iterate on ideas quickly and cheaply but also signaled to young people and pharmacists that they could provide honest feedback, even if it meant dramatically changing the approach. When refined and polished prototypes are presented, participants are more likely to hold back critical feedback if they believe much of the design is already fixed.^22^

**Figure:**
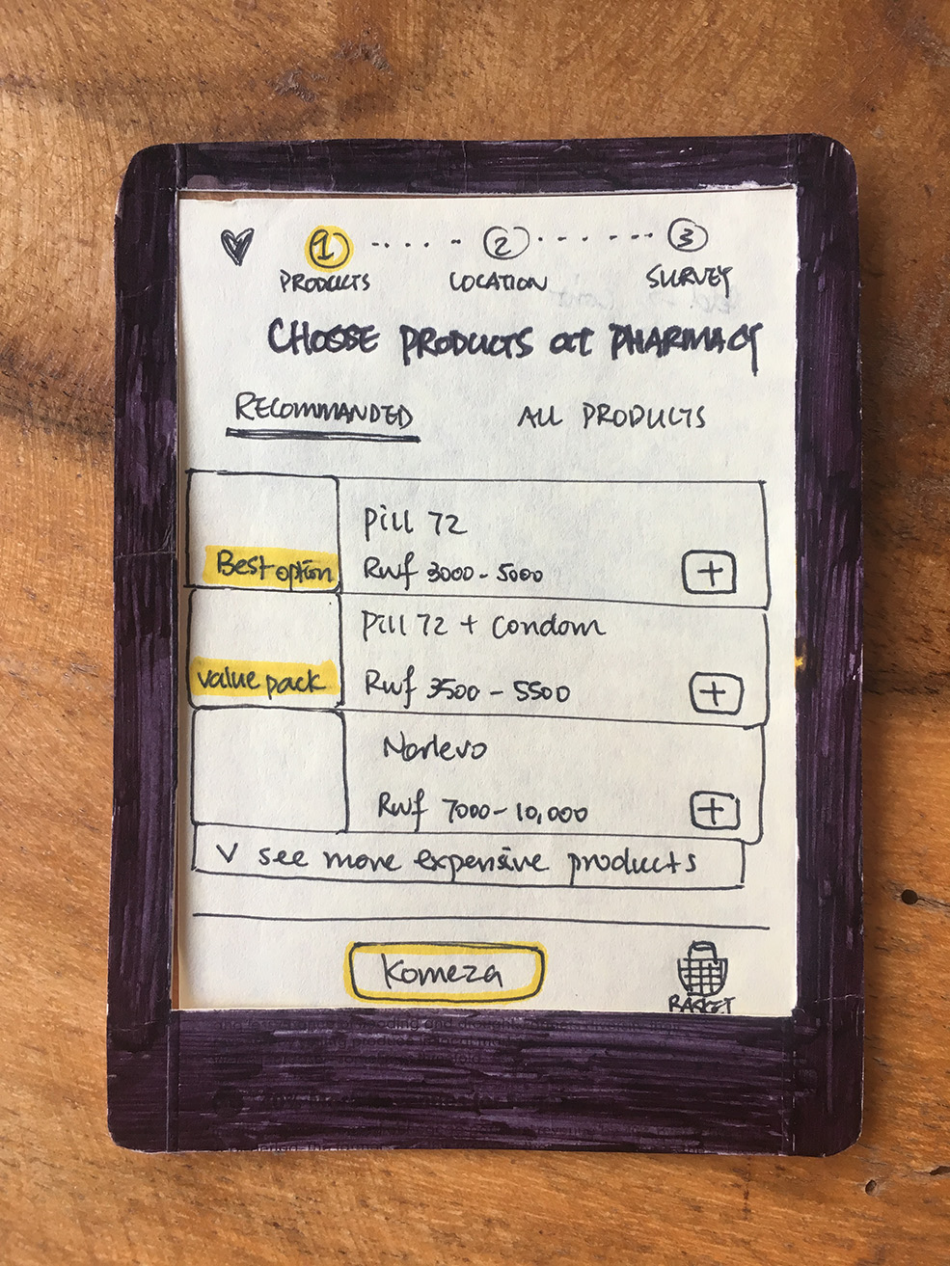
Rough prototype of Shop interface. © 2018 Caroline Wong/YLabs

**TABLE 3.** Prototyping Methods Used to Develop CyberRwanda

**Methods of sharing prototypes used**	**Description**
Paper prototyping: used to develop the initial design platform and subsequent iterations, content, and marketing materials (content, UI/UX, social media marketing strategy, promotional materials)	Paper and cardboard prototypes are used or co-created to create a quick but interactive physical representation of the product or experience.
Role play: Used in prototyping the end-to-end ordering experience	Participants are assigned certain scenarios and roles to help designers understand the interactions through observing the improvised scenario-specific performances.
Storyboarding: Used in prototyping workshops with pharmacists to walk through the potential value of the platform and pain points	Drawings or images displayed in sequence for pre-visualizing a new experience or service (e.g., a clinic visit).
Low- and high-fidelity interactive prototyping/digital prototyping: Used in the final stages of prototyping of the digital platform	Participants interact with an interactive prototype that has the look and feel of the final product.
“Wizard of Oz”: used to test a motorcycle taxi delivery service prototype	Participants interact with a digital product that appears to be computer-driven, when in fact responses are human-controlled. This method may intersect with others listed here.
**Methods of eliciting feedback and preferences**	**Purpose**
Ideation and co-creation: used to design and iterate on the cast of CyberRwanda webcomic characters, as well as developing storyline and content)	Participants generate entirely or partly new ideas for prototypes to test based on a design brief.
Elicitation: commonly used at the end of a prototyping session after showing multiple concepts	Asking the participants directly what improvements can be made to the prototypes to better reflect their needs and desires, what they prefer, and why.
Ranking: used to rank preferred channels of communication for young people to inform CyberRwanda marketing (e.g., posters, radio ads/shows, referral cards, and SMS alerts)	Participants vote and/or rank their preferred prototypes and top concerns.
Pre-/post-survey: this was used to evaluate knowledge improvement after interacting with an early prototype	Use surveys to evaluate change in specific indicators pre- and post-interaction with prototypes. The level of rigor may vary depending on the needs and the phase of the project. In early stages when testing low-fidelity, “rough” prototypes, these may help in rapidly deciding on prototype selection, but the sample size may not be powered appropriately. During the pilot, and implementation, and sometimes for live prototyping, an adequately powered sample is used.

Prototyping was conducted in 2 phases to enable the team to iterate ideas and develop increasingly high-fidelity prototypes based on learnings from the previous phase. Prototypes were tested and assessed based on predefined criteria that included desirability for participants, cultural acceptability, and feasibility. We used research questions to assess these factors during the prototyping phase and the subsequent ways in which they informed pivot points for the intervention design (Box 1).

BOX 1 **Research Questions Used to Develop Prototypes of CyberRwanda**

**Do the themes and tone of the educational content resonate with young people?**

Our initial prototypes for youth and providers included a virtual nurse/educator that answered questions through real-life stories. Through prototyping this idea with young people, they shared a preference for combining information about future planning and employment with the health-focused content. Parents, who reported concerns about their children being exposed to the often-taboo topic of family planning (FP)/reproductive health (RH), responded positively to prototypes presenting CyberRwanda as a guide to future health success, integrating employment-related content. Therefore, the final CyberRwanda curriculum was changed to reflect these preferences. The content on CyberRwanda today contains RH and FP content but also integrates other key aspects that young people care about, such as finding a job, goal setting, and economic empowerment. The content role models conversations between young people and parents, between parents, and between young people and providers and provides examples of how to navigate conversations on sensitive topics.

**How do we ensure accessibility and affordability through the delivery of the CyberRwanda program via various digital channels (i.e., SMS, native app, etc.)?**

Via focus groups and interviews, the team carefully mapped young people’s access to and use of different digital delivery channels such as personal or shared mobile devices and use of different social media and printed media channels. Through this research and low-fidelity prototyping (Table 3), youth expressed that they rarely download native apps and had concerns about the costs of data and memory use associated with using a native app. Furthermore, young people shared that they were using computer labs at the youth center to look for jobs and access information through online resources. They wanted CyberRwanda to be among the resources they could easily access using those computers. Therefore, the team pivoted the design to a web app that could be more readily accessed on any phone, tablet, or computer. This finding also expanded the implementation model to focus on loading CyberRwanda on tablets in youth centers along with schools. However, some schools and youth centers are in areas where Internet connectivity is not reliable, which delays the loading of content for users. As a backup, the team also developed a native app that is installed on tablets at schools and youth centers. With the native app, the youth at the schools and youth centers can access CyberRwanda content offline without needing to rely on the Internet.

**What would encourage young people to provide accurate information when accessing over-the-counter contraceptive methods?**

During design research, pharmacists expressed concerns about young people not honestly answering medically relevant questions when aiming to purchase contraceptive products. Young people were concerned about their privacy when requesting services in front of other customers. Youth reported pretending to purchase products for someone else to avoid judgment and potentially uncomfortable questions. In turn, pharmacy staff had concerns that young people were purchasing products without the necessary information to use them safely.

Initial prototypes were developed to explore how privacy might be improved in pharmacy settings. Initially, a “screener” survey prototype was explored. Upon arrival, the pharmacy staff directed young people to a paper survey including all medically relevant questions. Young people were concerned that other customers would be able to view private details while they were completing the form. Using the learnings from this prototype, the shop portion of the CyberRwanda platform now includes an online questionnaire with medically relevant questions that the user completes before purchasing a product. Depending on their answers, the platform allows them to proceed with the order or suggests a better alternative.

### Product and Content Development Through User Testing

Building on lessons from the previous stages of work, the team proceeded to develop the final digital intervention design. Although at this stage the intervention was more fully refined, additional testing and iteration with young people and pharmacists were conducted during all stages of content development, web application user experience design, service design development, and visual design. Product refinement and testing during this phase enabled the team to improve user experience and identify any potential barriers to use through testing the web app flow and function, brand, look and feel.

To develop the narrative and factual content in preparation for the RCT, a team comprised a narrative content writer, social norm and FP experts, and a behavioral scientist mapped the behavioral outcomes to the theory of planned behavior. The team determined how best to communicate key messages throughout the content based on an intervention mapping protocol for integrating theory and evidence-based behavior change methods into an intervention.^23^

### Pilot

In preparation for the RCT launch across 60 schools and 75 pharmacies in 8 districts, an 8-week pilot was conducted to finalize service design and implementation models, baseline data collection tools, and training. The pilot, conducted across 4 schools, 1 youth center, and 8 pharmacies, enabled the team to test every component of the web app and implementation models before a large-scale launch. Through post-pilot in-depth interviews and focus groups with 158 stakeholders and students, data from Google Analytics, and pilot baseline test data, the team gained critical learning and iterated appropriately. Learning from the pilot informed the need to provide more intervention touch points for pharmacists and pharmacy staff as primary users, to increase the number of tablets provided at each school, to refine the school recruitment and sensitization strategy, and to improve the user experience of the shop and the site as a whole (outlined in the Results section).

### Adaptive Implementation and Evaluation

CyberRwanda is entering into a 2-year implementation and evaluation phase, in which the same HCD methodology used to design CyberRwanda will be applied as the team iterates the design of the web app and adds features and content. This will include working with young people to develop all new narrative content and validating it before it is finalized, analyzing data generated by Google Analytics to understand which pages and content young people respond to the most, conducting focus group discussions with youth and teachers on additional topics and features that are needed, and working with parents and other stakeholders to support usage of CyberRwanda in schools and at home. From 2018 to now, the team continues to use HCD to iterate, test, and evolve CyberRwanda’s product and implementation model, with the participation of an additional 400 stakeholders.

Throughout the design and adaptive implementation process, the team prioritized several special considerations when applying HCD approaches to designing with youth (Box 2).

BOX 2 **Methods Used to Safely Design With Youth**

**Training youth designers**. Using a predominantly youth-led or youth-driven approach (Table 1), young Rwandans were trained as youth researchers to conduct human-centered design (HCD) sessions and most sessions, including user testing and development of all content, were led by youth. Trained youth designers were compensated in line with national salary benchmarks and not expected to volunteer their services for free.

**Creating safe spaces**. The design team used multiple techniques to support the development of a safe space for youth to engage by incorporating principles of trauma-informed care and led icebreaker activities designed to address the inherent power differential between researcher and participants. Less taboo topics were leveraged as “gateway” activities, and the team took steps to ensure that the session was valuable to youth by providing activities of interest and use to them (such as follow-up question and answer sessions). All young people were compensated for their time and participation.

**Ensuring comprehensive safeguarding protocols**. Several precautions were taken to ensure that child and youth safeguarding measures were in place. All staff were background checked and trained on child protection and safeguarding and followed robust child protection policies. Staff never conducted sessions alone with young people.

**Conducting comprehensive risk mitigation assessments**. Sensitivities and stigma surrounding reproductive health means prototyping with youth could potentially pose a social or safeguarding risk to them. Risk analysis and mitigation planning were conducted before prototyping, and prototypes were also tested with parents, teachers, and community leaders for acceptability and to help inform risk estimation before the pilot.

**Ensuring ethical use of participant images and data**. All young people provided written consent or assent with adult consent for their participation in the HCD process. Images were only taken and/or used with written consent from the young person. No images of youths aged younger than 18 years were used in program materials.

## RESULTS

From 2016 to the present, more than 1,000 Rwandan youth, caregivers, teachers, health care providers, and government stakeholders have been engaged in the design and evolution of CyberRwanda to answer the question, “How might we leverage technology to address teen pregnancy and unemployment among urban youth?” The HCD process resulted in a digital behavior change intervention designed with 2 main users in mind: urban and peri-urban young people and pharmacy staff (nurses and head pharmacists).

**Figure:**
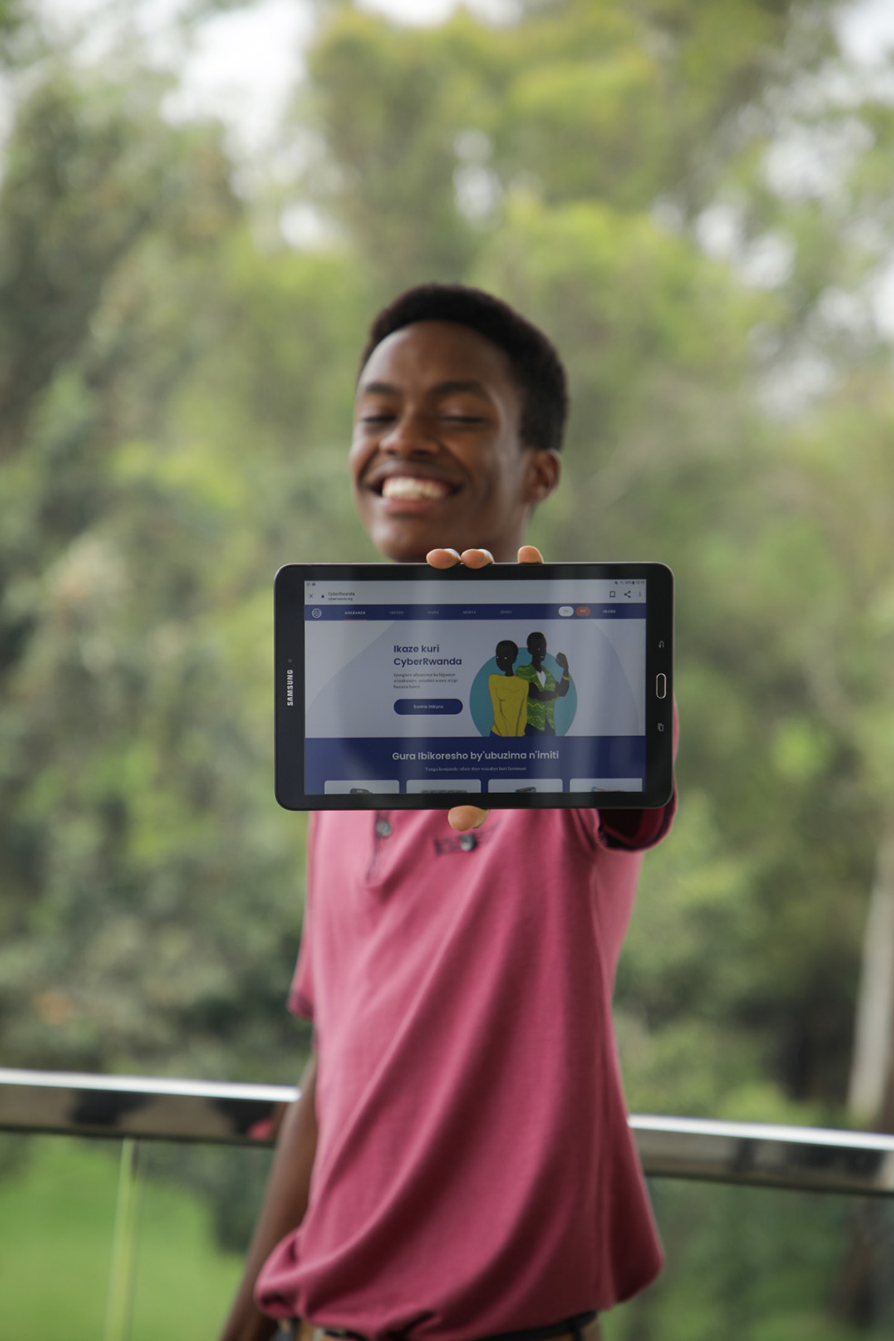
CyberRwanda web app loaded on a tablet. © 2020 Oscar Muhire/AfricaMile

The HCD process resulted in a digital behavior change intervention designed with 2 main users in mind: urban and peri-urban young people and pharmacy staff.

For young people, the web and future native app includes 3 sections:

1. **Stories.** Interactive behavior change webcomic series, with stories featuring a cast of characters designed with young people.
2. **Learn.** A robust library of young people’s priority questions, with more than 200 (and growing) frequently asked questions and a directory to help youth locate both public and private health care facilities and pharmacies in their area.
3. **Shop.** Young people can discreetly and privately order, purchase, and pick up certain pharmaceuticals and other health products at the CyberRwanda pharmacy of their choice. Location and pricing are transparent on the site.

CyberRwanda will be available to participating schools and youth centers where the intervention is made available on online tablets. Youth may also access CyberRwanda on their personal devices and school computers, where available.

For pharmacy staff, CyberRwanda is building a network of private pharmacies to support the provision of high-quality, youth-friendly care. Pharmacies who join this network have access to:

- **Training:** In-person and virtual training to CyberRwanda pharmacy staff. The 1-day in-person training for either the staff nurse or pharmacists covers key topics such as youth-friendly service provision, FP methods, and how to use the CyberRwanda platform and pharmacy portal. Providers who attend the CyberRwanda training will receive continuing professional development credits through accreditation by the National Pharmacy Council. A more condensed accredited version of the training is available virtually for all other pharmacy staff.
- **Portal:** Pharmacies access the online CyberRwanda pharmacy portal that enables them to add and update health products and pricing online as part of the Shop. When youth order products from the pharmacy of their choice, pharmacy staff can build relationships with young people in their communities.
- **Additional support:** The CyberRwanda team provides weekly support to pharmacies to ensure that they can use the portal and receive orders and to identify additional training areas and needs.

Implementation will be accompanied by a 2-year impact evaluation, consisting of a 3-arm RCT in schools, serial cross-sectional study in youth centers, qualitative studies, and a cost- effectiveness evaluation across 60 schools and 9 youth centers in 8 districts in Rwanda (sample at baseline n=6,082; 2,953 male; 3,129 female).^6^ The 3 arms of the RCT are: (1) “self-service” model: schools will be provided with tablets, a hotspot, and trained school ambassadors who promote CyberRwanda; (2) “facilitated” model: schools receive the same as the self-service model, plus structured activities held in a CyberRwanda club, where trained peers support youth to explore CyberRwanda content in a group setting with discussion and reflection; (3) control: schools without any form of the intervention. In addition to being implemented in the schools participating in the RCT, the self-service model will also be implemented in 9 district-level youth centers and 4 pilot schools, totaling an additional 13 implementation sites.

Table 4 shows key findings during the design process and the resulting major changes that were made to the intervention design. Using HCD as an end-to-end approach resulted in several critical process learnings that were foundational to the eventual design and implementation of CyberRwanda.

**TABLE 4.** Key Findings and Iterations to Intervention Design of CyberRwanda

**Key Finding**	**Iteration to Intervention Design**
**Supply-side barriers to quality care**	
Youth often saw health care providers as “gatekeepers” to accessing methods and were afraid about the questions they might be asked at health care facilities	We pivoted the platform away from simply linking to clinics to providing direct-to-consumer products and information directly in the hands of youth to reduce barriers associated with gatekeeping. Medically relevant screening is conducted at point of order to minimize bias-related barriers to FP/RH care and expedite the interaction between provider and youth. Youth can choose from a selection of pharmacists with transparent pricing.
Youth strongly preferred pharmacies as a point of access for contraception due to expediency and privacy	Deliver products and services via pharmacies: Young people’s priority concerns for privacy and expediency informed the pivot to supplying products via pharmacies instead of clinics, as originally planned. The platform also links to all public clinics in the intervention districts for longer-term methods.
Pharmacists lacked confidence in addressing young peoples’ SRH needs and had knowledge gaps on FP	Designed tailored training modules with and for pharmacists and pharmacy staff to address common misinformation, provide training on adolescent-friendly care, and support self-identification of provider bias to help providers improve access to care for adolescents.
**Demand-side barriers to quality care**	
Program lacked content on economic opportunity and job readiness, making it less appealing to youth and less acceptable to parents	The webcomic stories and FAQs now include content on preparing for employment and address gender stereotypes on careers. The site links to job search resources. The site tagline is “learn about relationships, your body, and your future.”
Boys had clearly expressed needs for information on health, relationships, and reproductive health	Content, characters, and the direct-to-consumer platform were codesigned and implemented with and for young men.
Youth lacked access to trusted, nonjudgmental information in accessible language they could understand	Characters were designed with youth to represent trusted personas for young men, women, and parents. A webcomic format was introduced to deliver information in a compelling, locally relevant way, focused on addressing key behavioral barriers and social and environmental norms that stigmatized access to FP/RH services. All content was reviewed by gender and behavior change and validated by youth. Finally, it was approved by the National Health Communications Committee to ensure alignment with national guidelines.
Young people were primarily concerned with lack of privacy and confidentiality in accessing information and services	To minimize barriers to engagement and allay fears about confidentiality, no identifiable data were collected from youth, even though this limited the ability to track individual user engagement. All data from the CyberRwanda platform were gathered and stored in compliance with General Data Protection Regulation guidelines. The shop was designed so that users can order without supplying personal information, without needing to request the product in person from the pharmacist. An order code is provided, which is needed to collect their order, with an optional SMS to their phone.
Given low levels of knowledge on FP/RH, youth often seek help for a specific problem rather than a specific product	In the shop, we pivoted to include both products for quick access for informed users and added common scenarios as another pathway to access products on the platform for naïve users or users experiencing a crisis (e.g., “I had unprotected sex.”). When a user clicks on those scenarios, they access essential information and recommendations of products and additional services available.

Abbreviations: FAQs, frequently asked questions; FP, family planning, RH, reproductive health; SMS, short message service.

### Expanding the Audience of Focus

CyberRwanda’s original demographic focused on adolescent girls. However, design research sessions revealed that young men had limited knowledge about FP/RH and had few trusted sources of information on the topic, though they were often the most influential driver of girls’ FP decision making. Young men lacked the confidence to access condoms and shared the same concerns about privacy when seeking care at a clinic. Based on these insights, the project team expanded the project scope to include information, topics, webcomic characters, and delivery channels that appealed to young men as well as girls.

*I had a perception that when a girl gets pregnant, there’s nothing to do other than wait to have a child. Or when a girl has forgotten to take emergency contraceptive after 72 hours after unsafe sex, I thought that that it is over; if you attempt to abort, you can die.* —Male secondary school student, 18 years old, interviewed during the pilot

*As young people, we fear being seen by a neighbor at the shop when you have to buy a contraceptive, you are shy that you are judged of what you are going to use the condom for*.—Male secondary school student, 18 years old, interviewed during the pilot

Findings from the pilot evaluation revealed that pharmacists and pharmacy staff needed to be seen as primary users. By doing so, the team further refined CyberRwanda’s role in working with pharmacists by expanding the focus audience to not only the head pharmacist in a pharmacy but also the nurse as young people trusted both but went to each for different reasons. Additionally, the team revised the training tools in partnership with pharmacists to ensure that content and the training approach addressed pharmacists’ most common information gaps, was acceptable and accessible to them, and also leveraged their motivations to better serve adolescents. The training has been accredited with continuing professional development credits by the National Pharmacy Council to increase the value of and trust in the program to participating pharmacists.

### Designing Educational Content

Extensive prototyping with a broad range of young people revealed how to design educational content for digital inclusion, different learning styles, and multiple literacy levels. The early and continued engagement of a spectrum of heterogeneous young people regarding digital access, literacy, educational, and socioeconomic status informed the development of an inclusive and accessible intervention. Based on youth preferences for shorter content, the intervention pivoted to a less text-dependent webcomic format delivered in both Kinyarwanda and English to accommodate different levels of literacy and support visual learners. In response to input from youth and parents on what makes a trusted health advisor, characters who deliver the content through stories were codesigned to represent clinic providers, aspirational older peers, and parents themselves. Given low levels of understanding about puberty, menstruation, and consent, additional topics were added.

Finally, there is little evidence on the level of in-person facilitation needed to implement digital interventions in school settings. We had found that youth were familiar with and actively participated in school health clubs. Therefore, we decided to compare a facilitated model, building on this existing school club culture to facilitate group and peer-driven learning (especially for less tech-savvy users), with a self-service model that supports potentially more private, self-directed learning. During the pilot in 3 schools, a higher percentage of students reported using CyberRwanda at the self-service school as compared to the facilitated schools (95% vs. 45% of students, respectively). Qualitative interviews with staff and students informed a redesign of the training and onboarding of teachers to reduce gatekeeping of tablets and improve access to CyberRwanda in both models during implementation.

Comparing a facilitated and self-service model for in school settings, a higher percentage of students reported using CyberRwanda at the self-service school.

Given variable levels of digital access especially between rural and urban youth, CyberRwanda is delivered via tablets in schools and youth centers as well as online, rather than relying solely on youth access to smartphones. Video instructions were designed to help youth with low literacy and low confidence using online ordering to easily navigate the shop function of the platform.

*I was interested by the part called ‘Shop.’ You can order contraceptive products using this program and get the products without the people knowing what you ordered. I like this part so much more than other parts, but I didn’t order anything.* —Female secondary school student, 14 years old, interviewed in pilot study

### Accessing Health Care Services

Design research and prototyping changed the interventions’ health care access points to cater to youths’ preferred service delivery points of pharmacies. In the early design stages, the team’s initial assumption had been that intervention would refer youth to primary health care clinics for FP/RH service delivery. However, during design research, young people shared fears of seeking health care services at the clinic for fear of being seen by fellow community members, which clinic staff confirmed.

*Youth don’t want to come to the clinic because they are afraid of being seen by their parent or someone they know.* —Nurse, Kigali

Both pharmacists and youth reported that they commonly opt to use emergency contraception to prevent pregnancy after an “accident” (unprotected sex), as youth perceive this as more socially acceptable than admitting to “planned” premarital sex, which is often stigmatized. Pharmacists reported seeing the same youth return repeatedly for emergency contraception after multiple “accidents,” suggesting young people are using emergency contraception as a regular method to avoid provider stigma. Given that youth can readily access emergency contraception at a pharmacy with minimal risk of exposure, they often do not seek longer-term contraception at clinics. In fact, youth are so reluctant to visit clinics, they prefer to pay for products or services (e.g., pregnancy tests and emergency contraception) at the pharmacy that could be accessed for a much lower cost at the clinic.

Youth had a strong preference for receiving FP/RH services at the pharmacy, valuing the expedient, confidential, and private method of service delivery. However, pharmacists were often reluctant to provide contraceptive products to unmarried adolescents, lacked private spaces for counseling, and often had inaccurate knowledge about FP. These findings informed a pivot toward a direct-to-consumer platform providing streamlined access to and online screening for health products supplied by a network of trained pharmacists, with signposting to clinics for longer-acting FP and other RH care.

A shift to a direct-to-consumer platform provided streamlined access to and online screening for health products supplied by a network of trained pharmacists.

*I struggle to counsel my customers on FP because our pharmacy has no private space.* —Pharmacist

*I feel more comfortable prescribing the oral contraceptive pill to a married woman because waking up to her husband on a daily basis will remind her to take the pill. Unmarried women don’t have this kind of reminder.* —Pharmacist

### Designing for Accessibility

User testing revealed that despite rapid increases in technology ownership and use, for many Rwandan youth, especially those with lower incomes, using a smartphone or tablet is a new skill. CyberRwanda had to be designed to be accessible and usable for 2 distinct groups: young people who had never used a smartphone before with minimal digital literacy and those with prior use and greater fluency in using tablets/smartphones. User testing allowed the team to observe where young people became confused or lost interest and allowed them to make design changes that made the user experience as streamlined and intuitive as possible. It was critical to engage those with low or no digital literacy in user testing, as features and navigations that are often assumed to be straightforward by young people with greater technological capabilities often become standard design specifications in digital solutions. Engaging those with lower digital literacy in user testing allowed the product team to more carefully integrate user flows that were more intuitive for an audience with minimal digital capabilities or experience. In Rwanda, online ordering is relatively new, even for experienced technology users. For the shop feature, where youth can order health products, prototyping informed the addition of written simple instructions to onboard new users, design elements to make action and navigation items easily noticeable, progress indicators, and simplification and shortening of the order process overall. In response to youth requests, we developed 2 instructional videos: one to help youth understand the CyberRwanda features and a second to help them navigate an unfamiliar process of online ordering and product collection at the pharmacy.

## IMPLICATIONS FOR FUTURE PRACTICE AND RESEARCH

The HCD process, driven and led by youth, was used to understand the complex user needs regarding FP/RH and promoted the routine testing, learning, and refinement of initial ideas. It paved a supported pathway toward implementation and evaluation by ensuring that the ultimate intervention was desirable among many key stakeholders, feasible for nationwide implementation and scale. Some implications for future practice and research follow.

## HCD Offers Foundational Methods and Mindsets to Support Continued Iteration Throughout Implementation

Using an adaptive implementation approach, the findings from routine user testing and the impact evaluation will inform iterations to the program and digital product design throughout the project timeline. Applying HCD during the implementation and evaluation stages of the intervention lends itself well to adaptive learning and iteration; allowing for new information to be continuously introduced that promotes rapid and informed decision making about which intervention elements need to be strengthened or abandoned.^24^ To achieve these aims, CyberRwanda will operate on a product release cycle where every 3 months the team reviews historical data to assess what new features to implement and how to make improvements. Monitoring data are collected from intervention sites and pharmacies routinely to identify areas of improvement and correct any issues that are disrupting site use. Qualitative interviews with participants are conducted on a routine basis to understand and supplement data gathered from quantitative data collection efforts. By taking this approach, HCD may help to support the long-term sustainability and cost-efficiency of the intervention by assuming a gradual approach to product launch and scale and addressing implementation challenges early and often, rather than relying solely on endline results.^25^

HCD may help to support the long-term sustainability and cost-efficiency of the intervention by addressing implementation challenges early and often, rather than relying solely on endline results.

### Iterative and Equity-Centered Approaches to Design Can Allow for the Inclusion of New Technology Users in Digital Initiatives

Digital self-care or direct-to-consumer approaches like CyberRwanda have the potential to offer the privacy that young people prioritize in FP/RH service delivery, yet require careful design to ensure usability by a broad range of users.^26^^,^^27^ User testing of early product versions revealed usability challenges that were addressed and retested in iterative cycles. This iterative approach to design and testing allowed the team to test assumptions and observe which features needed improvement to enhance the user experience and flow. To ensure digital equity and inclusion, particularly among those with limited or no access to technology, the team was intentional about sampling a diverse spectrum of participants regarding socioeconomic status, educational status, social vulnerabilities, and those from rural, urban, and peri-urban contexts. Recruitment of diverse participants was a recurring challenge during this project, and appropriate time and funding are needed to support inclusive recruitment. This article, along with other international digital health guidance from the World Health Organization,^28^^,^^29^ offers methods to inform a final product design and implementation model that is inclusive of diverse user groups. The forthcoming RCT will provide much-needed data on uptake and sustained use of the platform, disaggregated by age and gender among diverse youth populations.

### Engaging Youth Throughout the Design Process Requires Special Considerations, Particularly When Designing for Taboo Topics

Given the unique set of vulnerabilities and inherent power dynamics present when designing with youth on sensitive sexual and RH topics, there is a critical need for design teams to identify and plan for any risks that would compromise youths’ safety or compromise the quality of their engagement. The importance of upholding the continued safety and dignity of young people in the HCD process has gained special attention within the adolescent sexual and RH and HCD community of practice. Recently, several actors in the HCD community released a set of guiding principles for the safe and ethical engagement of youth during adolescent and sexual RH program design.^12^ In line with these guiding principles, the team took extra care to address power differentials between young people and program teams. Throughout CyberRwanda’s design phase, the project team sought to ensure the safety, dignity, and ethical participation of youth. During the initial design phases, the project team leveraged gateway activities focused on popular culture, future aspirations, and leisure activities as gateway topics to explore more taboo topics. Doing so enabled the team to provide a safe space for open and generative conversation with youth participants and created a foundation to explore sensitive sexual health topics. The emphasis on safeguarding and protection of youth was considered essential throughout all phases of the HCD process to protect young people’s safety and wellbeing. To maintain the safe and ethical participation of youth beyond the design phase throughout implementation, there was a clear need to understand what framing would be acceptable to parents, as key influencers and decision makers in their children's lives. The visioning of CyberRwanda as a guide to future success was co-created through the sessions with parents and has been a foundational framing for the project to date.

## CONCLUSION

Using HCD as an end-to-end approach was instrumental in guiding the team to learn, test, and refine the intervention before implementation and evaluation. It provided a natural approach to center young peoples’ needs, aspirations, and perspectives into the intervention design by having youth lead design research, prototyping, and data collection efforts with their peers. The process toward designing and implementing CyberRwanda has demonstrated the value of supporting youth-driven or youth-led HCD approaches that shift decision-making power to young people through funding, mentorship, and training. Furthermore, the HCD process provided a structure to integrate and blend technical and design expertise to ensure the intervention content was grounded in the adolescent health evidence and best practice, and the visual and product design was developed with user needs, access capabilities, and preferences in mind. Through trial and learning through continuous data collection and iteration, HCD informed a product and service design model that prioritizes digital equity. Through continuous learning and refinement, CyberRwanda’s hybrid implementation model was designed to meet the full spectrum of digital access and literacy and to reach vulnerable youth without access to technology. CyberRwanda’s forthcoming RCT will generate critical new data in fields of digital health for adolescents and social and behavior change communication. By assessing the difference between young people in the self-service and facilitated arms, findings will reveal the impact of a digital intervention as a stand-alone (self-service model) versus that with a person-to-person component (facilitated model). These findings will demonstrate which programmatic components lead to behavior change and how best to implement digital interventions for youth.

## Translation

**En Français**

**Utiliser le Design Centré sur L’humain Pour Développer, Lancer et Évaluer Une Plateforme Nationale de Santé Numérique Pour Améliorer la Santé Reproductive Pour la Jeunesse du Rwanda**

**Principales constatations**

**Utiliser l’approche du design centré sur l’humain durant la conception et la phase de la mise en œuvre** avec les jeunes et les parties intéressées a conduit à des pivots majeurs du produit, de la mise en œuvre et l’évaluation de la conception d’un produit de santé numérique pour la jeunesse du Rwanda.

En utilisant la processus du design centré sur l’humain pour développer CyberRwanda, il était crucial d’inclure une population diversifiée de jeunes gens et des parties intéressées, tous en tant que collaborateurs et leaders dans le processus d'une conception axée sur les jeunes.

Donner la priorité à un accès équitable a conduit à la conception des interventions pour les utilisateurs avec ou sans leurs dispositifs mobiles et en variant les niveaux d’alphabétisation numérique et de l’expérience avec la plateforme numérique.

**Les principales implications**:

Les organisations qui développent les produits de la santé numérique pour les jeunes devraient considérer une approche de bout en bout du design centré sur l’humain pour le modèle du développement du produit et de l’intervention.

Avec l'intérêt croissant et l’investissement dans les interventions en santé numérique, surtout ceux qui soutiennent les produits destinés directement aux consommateurs, les bailleurs devraient soutenir et encourager les fonds et le temps pour la co-conception, le développement et le prototypage avec le public désigné durant les phases de la conception et la mise en oeuvre.

Les professionnels du design centré sur l’humain centrés sur les jeunes ont besoin de s’assurer qu’il y a une bonne représentation de différents groupes de jeunes, prendre soin d’adresser le déséquilibre des pouvoirs parmi les jeunes et les concepteurs, et s’assurer qu’ils ont des politiques et pratiques solides en matière de sauvegarde.

**RÉSUMÉ**

**Contexte:** Un manque d’accès aux soins et à une information fondée sur les faits, impartiale et une planification familiale et de santé reproductive adaptée aux jeunes limitent la capacité aux jeunes d’éviter les grossesses non planifiées, le VIH et les maladies sexuellement transmissibles. Ceci menace leur santé et devient la cause majeure d’abandon scolaire, limite le bien-être des jeunes, leur potentiel pour l'avenir, et les opportunités d’emploi. Pour remédier à ces problèmes, YLabs a utilisé une approche de bout en bout du design centré sur l’humain en créant CyberRwanda, une plateforme numérique qui a pour objectif d’améliorer la santé et les moyens de subsistance des adolescents (âgés de 12 à 19 ans) au Rwanda.

**Concevoir pour le numérique avec les jeunes:** Depuis 2016 jusqu’en 2020, CyberRwanda a été conçu et testé en utilisant l’approche du design centré sur l’humain en partenariat avec plus de 1000 jeunes gens, parents, enseignants ainsi que les prestataires de soins publics et privés. Pendant la phase de la reconnaissance du problème, le design centré sur l’humain a révélé les croyances des participants, leurs préférences comportementales et leurs expériences en particulier avec la planification familiale et la santé reproductive ainsi que leur expérience en générale de la vie de tous les jours, les motivations, les aspirations, et les défis. Plusieurs phases notamment de prototypage analogues et numériques avec les jeunes et les décideurs clés ont été utilisées pour concevoir conjointement, tester et améliorer l’intervention pour la mise en œuvre.

**Les résultats:** CyberRwanda est une plateforme axée sur le consommateur où les adolescents peuvent apprendre des informations sur la santé appropriées à leur âge et qui renforcent leurs compétences à travers des histoires éducatives tournées vers le changement du comportement, une rubrique des questions fréquemment posées, commander en ligne des produits de santé et être connecté au réseau CyberRwanda des prestataires de soins privés et publiques qui ont été formés pour des soins adaptés aux adolescents.

**Les implications pour les futures recherches:** Le processus du design centré sur l’humain a abouti à des pivots significatifs dans la conception de la plateforme numérique et du modèle de la mise en œuvre. Utiliser le design centré sur l’humain a fourni une méthodologie structurée pour combiner l’expertise technique de la planification familiale et de la santé reproductive et l’expertise de la conception du produit pour concevoir conjointement et répéter une intervention de santé numérique avec et pour la jeunesse rwandaise.

**Peer Reviewed**
